# Splenic protection network revealed by transcriptome analysis in inactivated vaccine-immunized flounder (*Paralichthys olivaceus*) against *Edwardsiella tarda* infection

**DOI:** 10.3389/fimmu.2022.1058599

**Published:** 2022-11-09

**Authors:** Xiaoyan Wu, Jing Xing, Xiaoqian Tang, Xiuzhen Sheng, Heng Chi, Wenbin Zhan

**Affiliations:** Laboratory of Pathology and Immunology of Aquatic Animals, Key Laboratory of Mariculture, MOE, Ocean University of China, Qingdao, China

**Keywords:** immune protection, RNA-seq, spleen, *Paralichthys olivaceus*, *Edwardsiella tarda*

## Abstract

The protective immune response produced by fish after vaccination is crucial for vaccine effectiveness. Our previous studies have shown inactivated vaccine against *Edwardsiella tarda* can induce immune response in flounder (*Paralichthys olivaceus*). To elucidate the protective immune response at the genetic level, in this study, flounder was immunized with inactivated *E. tarda* for 5 weeks, and then they were challenged with *E. tarda*. The spleen was dissected at 7^th^ day post immunization, 1^st^ and 7^th^ day post challenge, respectively. Transcriptome analysis showed that average of 46 million clean reads were obtained per library, while percentage of clean reads being mapped to reference genome was more than 89% in all cases, which suggested good quality of samples. As for differentially expressed genes (DEGs) identification in inactivated *E. tarda* groups, at 7^th^ day post immunization, 1422 DEGs were identified and significantly enriched in innate immune-related pathways, such as Phagosome, Cell adhesion molecules and NF-kappa B signaling pathway; At 1^st^ post challenge day, 1210 DEGs were identified and enriched to Antigen processing and presentation and Cell adhesion molecules, indicating that the pathogen was rapidly recognized and delivered; At 7^th^ post challenge day, 1929 DEGs were identified, belonged to Toll-like receptor signaling pathway, Antigen processing and presentation, Th1 and Th2 cell differentiation and Th17 cell differentiation. Compared to 7^th^ post immunization day, 73 immune-associated DEGs were identified at 1^st^ post challenge day. Protein-protein interaction networks analysis revealed 11 hub genes (TLR7, TLR3, CXCR4, IFIH1, TLR8 etc), associated with recognition of pathogens and activation of innate immunity; while for 7^th^ post challenge day, 141 immune-associated DEGs were identified. 30 hub genes (IL6, STAT1, HSP90A.1, TLR7, IL12β etc) were associated with stimulation of lymphocyte differentiation and activation of cellular immunity. Ten immune-related genes were randomly selected for RT-qPCR validation at each time point. In conclusion, data revealed protection of flounder against *E. tarda* infection by inactivated vaccine is mediated *via* immediate recognition of pathogen and subsequently activation of cellular immunity. Results give new aspect for vaccine protection cascades, is good references for vaccine evaluation.

## Introduction

Vaccines induce protective immune responses in fish (1, 2). The immune protection involves the relative percentage survival (RPS), antibody production and T/B lymphocyte response against challenge etc (3–5). RPS has been widely used to assess vaccine protection (6, 7). Besides of the RPS, the production of antibodies is the strategy of vaccines. Antibodies specifically recognize and bind antigens, which promote phagocytosis and achieve clearance of pathogens (8, 9). T/B lymphocytes are essential component of the adaptive immune response for assessing vaccine protection (10, 11). Study in turbot (*Scophthalmus maximus*) immunized with inactivated bivalent vaccine (IVVah1) showed high RPS to *Vibrio anguillarum* and *Vibrio harveyi* infection were maintained from week 4 to week 8 post immunization. In addition to RPS, antibody levels showed a trend of increasing and then decreasing, peaking at weeks 2 and 3. After 8 weeks of immunization against the pathogen, antibodies increased within a week. This indicates that at the protein and individual level, turbot immunized with IVVah1 are protected against the pathogen (12). In the study of vaccine-immunized flounder (*Paralichthys olivaceus*) challenged with *Edwardsiella tarda*, antibodies increased significantly at week 3. The percentage of T lymphocytes peaked at day 7. The percentage of IgM^+^ B lymphocytes showed a trend of increasing and then decreasing, peaking at week 2. This suggested that inactivated vaccine enhanced protective humoral and cellular immune responses in fish after challenge (13). In addition to this, the immune protection of fish vaccines needs to be supported by fine grained networks and comprehensive data. This is urgently needed for the development and application of effective vaccines.

Flounder is valuable marine fish. In recent years, with the rapid development of intensive aquaculture, the incidence of disease outbreaks has been increasing, which seriously affects the culture of flounder (14). *E. tarda* is a gram-negative bacterium with intracellular parasitism (15). It causes high mortality and significant economic losses in flounder through invasion of epithelial cells, production of toxins, and evasion of phagocyte-mediated killing (16, 17). Safe and effective vaccines are essential for the control of bacterial diseases (18, 19), among which inactivated vaccines have become commercialized vaccine species (20). For elucidating the protective immunity mechanism of inactivated vaccines, it is essential to study the response of immune organs in pre-vaccinated flounder after infection (21). Similar to mammals, the spleen of fish is the main peripheral lymphoid organ (22). Here most of the antigens in the blood are captured and engulfed by macrophages. At the same time, it is also the site of aggregation of T and B lymphocytes for antigen presentation and initiation of adaptive immune responses. The spleen is an essential organ for resolving the immune response in fish (23–25).

Transcriptome sequencing analyzes gene expression dynamics at the level of individual transcripts, which can contribute to the resolution of immune-related genetic information and functional molecules (26, 27). RNA-Seq has been applied to analyze immune-related genes and signaling pathways in fish such as flounder, grass carp, large yellow croaker and turbot (28–31). The spleen red blood cells of flounder challenged with *E. tarda* were analyzed by transcriptome. 21 key genes were identified, mainly involved in antigen processing and presentation, pathogen recognition and inflammation (32). The phagosome pathway was activated in the head kidney of turbot inoculated with bivalent inactivated bacteria vaccine *Aeromonas salmonicida* and *Vibrio scophthalmi*. Antigenic peptide transport protein 1 (TAP1), complement fraction 3 (C3) and mannose receptor (MR) were significantly upregulated (33). However, it is often limited to the analysis of tissues such as head kidney, gill and blood at different time points after immunization or infection. This is still inadequate for supporting the molecular mechanisms of immune protection in fish. In addition, genes in organisms are functionally interconnected and together control the activities of the organism (34). For example, kidney from flounder infected with *E. tarda* was sequenced. Immune-related genes were used to construct protein-protein interaction networks (35). The identification of hub genes helps to understand the protective mechanism of the vaccine.

In this study, flounder were immunized with inactivated *E. tarda* vaccine for five weeks and then challenged with *E. tarda*. The infection status of flounder was evaluated. Spleens of flounder were sampled for transcriptome analysis at 7^th^ post immunization day, 1^st^ and 7^th^ post challenge day. RNA sequencing (RNA-Seq) was performed using the Illumina Novaseq6000 platform. The results showed that an average of 46 million clean reads were obtained per library, with clean reads accounting for more than 99% of raw reads and Q30 greater than 92% in all cases; while the percentage of clean reads being mapped to the reference genome was more than 89% in all cases. 1422, 1210 and 1929 differentially expressed genes (DEGs) were identified at 7^th^ post immunization, 1^st^ and 7^th^ post challenge day, respectively. Differential genes were annotated into Gene Ontology (GO) and Kyoto Encyclopedia of Genes and Genomes (KEGG) functional databases. These genes are involved in many immune processes, mainly Toll-like receptor signaling pathways, antigen processing and presentation, and Th1 and Th2 cell differentiation. At 1^st^ and 7^th^ post challenge day, respectively, 73 and 141 immune-related DEGs were used to construct protein-protein interaction network, predicting 11 and 30 hub genes involved in the immune response. This study provides the basis for further elucidation of the immune protection against bacterial infection in flounder immunized with inactivated vaccine.

## Materials and methods

### Experiment fish

Healthy flounder (*P. olivaceus*) (length range: 15-17 cm) were purchased from a farm in Rizhao, Shandong Province, China. The experimental fish were kept in the laboratory basement for two weeks, during which the water temperature was maintained at 21 ± 1°C in aerated seawater. The fish were fed with commercial pellets at 3% of body weight per day, and 1/3 of the seawater in the tanks was replaced. Before the experiment, flounders were randomly tested as free of pathogenic bacteria (36). Fish were anesthetized with tricaine methylate (MS-222, Sigma, USA) before tissue sampling. The treatment of fish in this study was approved by the Institutional Animal Care and Use Committee of the Ocean University of China (permit number: 20150101).

### Inactivated *Edwardsiella tarda* vaccine

*E. tarda* HC01090721 strain was isolated from the ascites of diseased flounder by researchers in our laboratory and stored in brain heart infusion (BHI) medium containing 15% glycerol at -80°C (37). The inactivated *E. tarda* vaccine was prepared by referring to the previous process (38). First, the conserved strains were inoculated on BHI solid medium by continuous scribing and cultured at 28°C for 24 h. Single colonies were inoculated on BHI liquid medium in oscillating incubator at 28°C and 180 rpm for expansion. After the bacteria had grown to the logarithmic growth phase, bacterial precipitates were obtained by centrifugation at 8000 g for 5 min and resuspended in sterilized 0.01 M phosphate-buffered saline (PBS; pH=7.4). The bacterial concentration was adjusted to 10× 10^9^ CFU/mL and inactivated by the adding 0.5% formalin (V/V) and shaking at 4°C for 72 h. The precipitate was collected by centrifugation at 8000 g for 5 min and resuspended with sterile PBS. 200 µL of inactivated bacteria were coated in BHI solid medium and incubated for 24 h at 28°C. The inactivation was proved to be successful if no colonies grew. The bacterial concentration was adjusted to 2.0×10^9^ CFU/mL and stored in a refrigerator at 4°C until use.

### Vaccination, challenge and sampling

**Figure 1:**
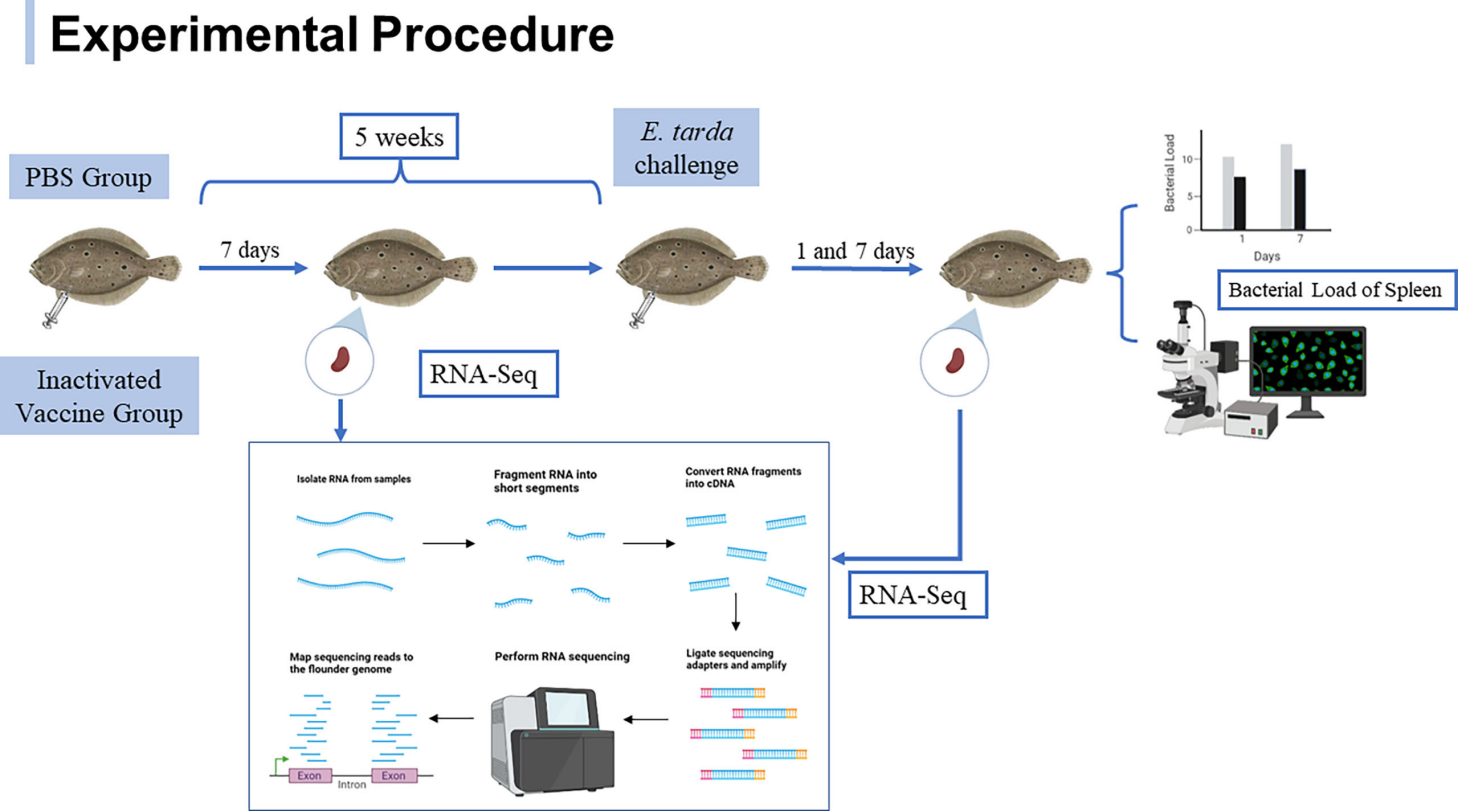
Experimental procedure of flounder after immunization with inactivated vaccine and subsequent *E. tarda* challenge.

Healthy flounder were randomly divided into two groups, the PBS group was injected intraperitoneally with 100 μL of PBS with complete Freund’s adjuvant (1:1) and the inactivated vaccine group was injected intraperitoneally with 100 μL of inactivated *E. tarda* with complete Freund’s adjuvant (1:1). After 5 weeks of immunization, 1.0 × 10^6^ CFU of *E. tarda* was inoculated for the challenge experiment. At 7^th^ day post immunization and 1^st^ and 7^th^ day post challenge, respectively, spleens from three fish in each group were randomly sampled and snap-frozen in liquid nitrogen for RNA extraction. Samples were marked: CS-7 (CS-7-1/CS-7-2/CS-7-3) for fish on the seventh day after immunization with PBS, IPS-7 (IPS -7-1/IPS -7-2/IPS-7-3) for fish on the seventh day after immunization with inactivated vaccine, ACS-1 (ACS-1-1/ACS-1-2/ACS-1-3) for fish on the first day after five weeks of PBS immunization with *E. tarda* infection, AIPS-1 (AIPS-1-1/AIPS-1-2/AIPS-1-3) for fish on the first day after five weeks of vaccine immunization with *E. tarda* infection, ACS-7 (ACS-7-1/ACS-7-2/ACS-7-3) for fish on the seventh day after five weeks of PBS immunization with *E. tarda* infection, AIPS-7 (AIPS-7-1/AIPS-7-2/AIPS-7-3) for fish on the seventh day after five weeks of vaccine immunization with *E. tarda* infection. To detect the infection status of sampled fish, remaining spleen tissue after challenge was immersed in RNALater (TaKaRa) for detection of bacterial load and also fully embedded in Tissue-Tek O.C.T. Compound (Sakura Finetek USA) and stored at -80°C for 4 h for immunofluorescence analysis. The specific experimental procedure is shown in **Figure 1**.

### RNA sample preparation, library construction and sequencing

For transcriptome analysis, 18 RNA-seq libraries were constructed using spleens. RNA was extracted from spleen using the Trizol according to the manufacturer’s instructions for RNA-seq analysis and subsequent RT-qPCR validation of transcriptomic data. RNA samples from spleens were subjected to RNase free agarose gel electrophoresis and Agilent 2100 Bioanalyzer for quality and integrity. The mRNA was enriched by Oligo(dT) beads and fragmented with fragmentation buffer. The mRNA was reverse transcribed into cDNA using the NEBNext Ultra RNA Library Prep Kit for Illumina (NEB, Ipswich, MA, USA). cDNA was purified using 1.8X Agencourt AMPure XP Beads, followed by end repair, the addition of base A, and ligation of sequencing adapters. Size selection of ligated fragments was performed by agarose gel electrophoresis and polymerase chain reaction (PCR). Sequencing of 18 cDNA libraries from spleen samples was performed on the Illumina Novaseq6000 platform at Gene Denovo Biotechnology Co. (Guangzhou, China).

### Data quality control, sequence alignment analysis and expression statistics

Raw reads of the spleen cDNA libraries contained low quality bases. The reads containing more than 10% unknown nucleotides and containing more than 50% low quality (Q-value ≤ 20) bases and containing adapter are removed according to FASTP (version 0.18.0) (39) to obtain high quality clean reads from the spleen cDNA libraries. The clean reads were aligned to the ribosomal RNA (rRNA) database using the short reads alignment tool bowtie2 (40), and the aligned ribosomal reads in the spleen cDNA libraries were removed. The retained clean reads were used for subsequent transcriptome analysis. The clean reads were mapped to the flounder reference genome (GenBank project accession: PRJNA344006) using HISAT2.2.4 (41). The mapped reads from 18 spleen cDNA libraries were assembled respectively by using StringTie v1.3.1 (42, 43). For each transcribed region, FPKM (fragment per kilobase of transcript per million mapped reads) values were calculated using RSEM (44) to quantify its expression abundance and variation.

### Analysis of differentially expressed genes, GO and KEGG functional enrichment

Splenic differentially expressed genes (DEGs) between the PBS and inactivated vaccine groups were analyzed using DESeq2 (45), and the screening criteria for differential genes were *p* ≤ 0.05 and expression fold change > 1.5. To further understand the biological functions of the genes, DEGs were mapped to terms in the GO database (http://www.geneontology.org/), and the number of genes per term was calculated. GO terms that were significantly enriched to differential genes compared to background genes were identified. KEGG (http://www.genome.jp/kegg/) is the main database on Pathway. Pathways that were significantly enriched by differential genes compared to background genes were obtained. A hypergeometric test was performed to identify significantly enriched GO terms and KEGG pathways using *p*-value ≤ 0.05 as the threshold.

### Protein-protein interaction network analysis

Venn diagram was used to demonstrate shared and unique DEGs for the three comparison groups after immunization and challenge. String (http://string-db.org) was applied to analyze protein-protein interactions that upregulate immune-related DEGs at 1^st^ and 7^th^ after challenge day, respectively. Cystoscape (V3.7.1) (https://cytoscape.org/) was used to visualize network file in which genes as nodes and interaction relationships as lines of the network. Studying the protein-protein interaction network helps to identify hub genes.

### Quantitative real-time polymerase chain reaction

To analyze the bacterial load in the spleen of flounder post *E. tarda* infection, spleen DNA was extracted using the TIANamp Marine Animals DNA Kit (Tiangen, Beijing, China) according to the manufacturer’s instructions. Specific primers (F: TAGGGAGGAAGGTGTGAA; R: CTCTAGCTTGCCAGTCTT) were used for the amplification of *E. tarda* gene fragments. Each sample was taken in triplicate. The bacterial load in the spleen (log_10_ *E. tarda* cells/0.1 g) was quantified according to a previously established standard curve (46).

To verify the reliability of the transcriptome data, immune-related genes were selected for qRT-PCR. RNA was adjusted to the concentration of 1 μg/μL using Nanodrop 8000 spectrophotometer (Thermo Fisher Scientific, Waltham, MA, USA). cDNA was obtained using HiScript III RT SuperMix for qPCR (Vazyme, Nanjing, China) for RNA reverse transcription. Gene specific primers were designed using Primer Premier 5.0 and the primer sequences are listed in **Table 1**. β-actin was used as internal control. The mRNA levels of spleens in the PBS group at each time were set to 1. qRT-PCR was performed using the LightCycler^®^ 480 II Real Time System (Roche, Basel, Switzerland). Each reaction system contained 10 μL of 2× Universal SYBR Green Fast qPCR Mix, 2 μL of cDNA template, 0.4 μL each of forward and reverse primers, and 7.2 μL of DEPC water. The reaction procedure was: pre-denaturation at 95°C for 3 min, 40 cycles including annealing at 95°C for 5 s and extension at 60°C for 30 s. Reactions were performed in triplicate. Gene expression levels were analyzed by the 2-^ΔΔ^Ct method.

**Table 1:** List of primers for qRT-PCR validation of differentially expressed genes.

Primer name	Forward primer (5’-3’)	Reverse primer (5’-3’)	GenBank Accession No.
IL1β	GAGATGGTGCGATTTCTGTTCTAC	ATGTTGAAGGTCTGGTAGCACTG	XM_020105656.1
CXCL12	TTGGTGTCGTTCTACCCTCAAC	TCTTCACCTTGTTGATGGCG	XM_020084401.1
IL21R	TACAATCTCACTCTGTCCCAACC	CTCCAGGTGAAGTGGTGTTGA	XM_020083502.1
RT1-B	GCAGCGTCTTTGACTTCTACCC	CCAGACTTGGGCATGTACTCC	XM_020108298.1
LPAR4	TAGTCTACCCTTTCCGTTCGC	TCACTGATATTCCTCCACCCAC	XM_020088202.1
JUN	TCCCACAACCACATGGATCAC	TCTCCATGTTGATGGGGGAA	XM_020109221.1
TLR7	GCTCAATAGGACCACAGTAACCA	CTAGCAATGGACAGGGTGAGG	XM_020089659.1
CCL19	GACATCAGCACAGGTTCCCA	GGATGGTGGCGTCGATAGAG	XM_020106263.1
IRF3	CAGTTCAGGGTGTCGGTGTACT	TCGGGTCAGTTTGGCTTGAG	XM_020107055.1
DHX58	GGAGTCGCTACACCGCTTCTA	GGCTCCCAGTCGTCAAACATC	XM_020094280.1
MAPK8	TTGACGCCTCCAAACGAATC	CCACTCATCCCAATCACTCACTT	XM_020092592.1
CXCL14	GATCAAACCCAAACACCCGTA	CCAGATGCGGAACCATTTG	XM_020086273.1
TLR8	CGTGATTGTGCTGCTGATGC	CTTGATTGTCCACCCTGACGA	XM_020089660.1
GADD45β	ACTGTCTGACTGTGGGCGTGTA	CCTGAAGCAGCGTGAAGTGG	XM_020109774.1
PPAP2B	CCCAGCATCACCTATCCTCAT	AGCGTACCCTGTAACACTCCC	XM_020083954.1
CCL25	GGGCACGTTAAGAGGATGAGG	TTGGCACAGACAGTCCGTTGT	XM_020081297.1
CCL20	AGGTTGTGGTGGATTGTTGTC	ATGATGCACAGGGTCTTCTCA	XM_020106265.1
HSP90α.1	CGCTGGTGGCTCCTTTACA	GCTTCTTCACAATCTCTTTGACTCT	XM_020091873.1
STAT1	GCAAGCAGAGTGCCAATGAGA	AAGGTGCCGGGACACTTGT	XM_020105149.1
IL5Rα	GTCACGGTGGAATCGTCAAGT	AAGGAATCGGAGGAAACAGAA	XM_020106780.1
CXCL8	AGTCTGAGCAATGGAGGAGTGA	CCAAGCACTTTATGACCCACG	XM_020100336.1
MAPK14A	TAATCATGCTGCTCGTCGGA	GCTGTTATCCGTTTGTCTGTGTC	XM_020089244.1

### Indirect immunofluorescence assay

To analyze the infection of the spleen by *E. tarda* as described previously (47), in brief, sections obtained using Cryostats (Leica CM1900) were fixed in cold acetone for 15 min. Sections were washed three times with phosphate-buffered saline containing 0.05% Tween-20 (PBST) and then sealed with 5% BSA at 37°C for 45 min. Sections were then washed three times with PBST for 5 min each and incubated with rabbit anti-*E. tarda* polyclonal antibody (1:2000) as primary antibody for 1 h. After washing away the unconjugated primary antibody, sections were incubated with Alexa Flour 488-conjugated goat anti-rabbit IgG (1:1000, Thermo Fisher Scientific, Waltham, MA, USA) for 45 min in the dark at 37 °C. After three washes with PBST, the sections were counterstained with DAPI on the nuclei and incubated for 15 min at room temperature in the dark. After blocking with glycerol, the green positive signal was observed under fluorescent microscope (Olympus DP70, Tokyo, Japan). Rabbit negative serum was used as a negative control (1:1000 diluted in PBS).

### Statistical analysis

All experiments were performed three times. Data were presented as mean ± standard deviation (SD). Data analysis was performed by SPSS 20.0 (IBM, Armonk, NY, USA). T-test was used to examine the differences between the PBS and inactivated vaccine groups. Differences were considered statistically significant when *p < 0.05. Graphs were plotted using GraphPad Prism 9 (Inc. San Diego, CA, USA).

## Results

### The load of *E. tarda* in the spleen of flounder after challenge

**Figure 2:**
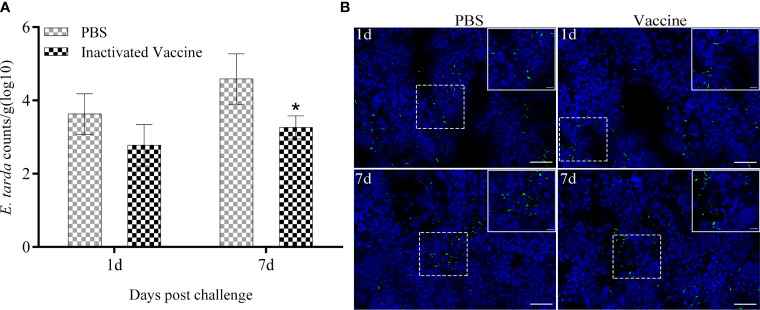
Bacterial load in the spleen of flounder sampled from the PBS and inactivated vaccine groups was determined by qRT-PCR at 1^st^ and 7^th^ post challenge day **(A)**. Values are shown as mean ± SD (N = 3). Asterisks on the bars represent statistical significance (*p < 0.05). Distribution of *E tarda* in the spleen **(B)** observed by indirect immunofluorescence (Bar = 20 um). The corresponding enlargement of the diagram in the dotted box is shown in the upper right of the image (Bar = 5 um). The green fluorescent represents signal of *E tarda*. Cell nuclei were stained with DAPI in blue.

The presence of *E. tarda* in spleen samples was characterized quantitatively by qRT-PCR and qualitatively by indirect immunofluorescence (**Figure 2**). At 1^st^ day post challenge, the bacterial loads in the spleen of PBS and inactivated vaccine groups showed small differences (*p* > 0.05). At 7^th^ day post challenge, the spleen bacterial loads were significantly higher in the PBS group than in the inactivated vaccine group (*p* < 0.05). A large amount of specific green fluorescence was present in PBS group, whereas a small amount of green fluorescence was present in the vaccine group.

### Transcriptome sequencing and read mapping of flounder

At 7^th^ day post immunization, 1^st^ and 7^th^ day post challenge, respectively, 18 libraries of spleen RNA from PBS and inactivated vaccine group flounders were sequenced. To ensure data quality, raw reads were quality controlled. The six groups produced an average of 43875712, 44863902, 43172840, 55465904, 40599382, and 52051965 clean reads, and the ratio of clean reads to raw reads was above 99%. Among these clean reads, the rates of Q20 and Q30 were above 97% and 92%, respectively, which indicated that the sequencing results were of good quality. Clean reads were mapped to the flounder reference genome at a rate of over 89% in all cases, with an average of 20,542 genes annotated to each library (**Table 2**). The raw transcriptome sequencing data were submitted to the Sequence Read Archive (SRA) in NCBI. The accession number is PRJNA870695.

**Table 2:** Summary of transcriptome data from splenic samples.

Sample	Raw_Data (bp)	Clean_Data (bp)	Clean reads (%)	Q20 (%)	Q30 (%)	Total_Mapped (%)	Total_Genes
**CS-7-1**	36028808	35901210	99.65	97.22	92.57	90.23	20208
**CS-7-2**	46360062	46205862	99.67	97.54	93.21	89.11	20297
**CS-7-3**	49674892	49520064	99.69	97.72	93.62	89.18	20517
**IPS-7-1**	43635786	43487332	99.66	97.82	93.84	90.96	21438
**IPS-7-2**	44625620	44476768	99.67	97.64	93.39	90.63	20512
**IPS-7-3**	46771046	46627606	99.69	97.73	93.65	90.47	20387
**ACS-1-1**	37501910	37371230	99.65	97.30	92.77	89.87	20420
**ACS-1-2**	45202050	45034962	99.63	97.10	92.36	90.35	20709
**ACS-1-3**	47535040	47112328	99.11	97.47	93.08	90.40	20363
**AIPS-1-1**	68575360	68364728	99.69	97.65	93.47	90.34	21007
**AIPS-1-2**	47851060	47700772	99.69	97.67	93.53	90.48	20541
**AIPS-1-3**	50503214	50332212	99.66	97.39	92.84	90.35	20839
**ACS-7-1**	36282856	36157128	99.65	97.18	92.51	90.15	19969
**ACS-7-2**	40904182	40764252	99.66	97.24	92.63	90.52	20401
**ACS-7-3**	45026286	44876766	99.67	97.31	92.72	90.52	20361
**AIPS-7-1**	47586686	47433578	99.68	97.67	93.50	90.72	20628
**AIPS-7-2**	56620094	56448808	99.70	97.60	93.31	89.92	20535
**AIPS-7-3**	52440960	52273510	99.68	97.66	93.49	89.85	20622

### Analysis of differentially expressed genes after immunization

**Figure 3:**
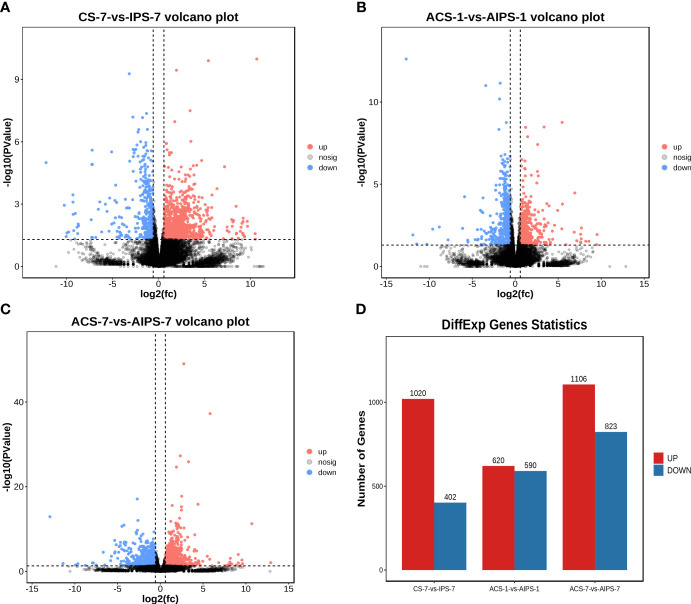
Volcano plots analysis of significantly different genes (DEGs) between samples from the PBS and inactivated vaccine groups at 7^th^ post immunization day **(A)** and 1^st^ **(B)** and 7^th^ **(C)** post challenge day. The red (expression up-regulated) and blue (expression down-regulated) points indicate differential expression of genes, and black points are no differences. Statistical plot of DEGs for the PBS and inactivated vaccine groups at three time points **(D)**.

The distribution of DEGs between the PBS and inactivated vaccine groups is shown using the volcano plot (**Figure 3A**). At 7^th^ day post immunization, there were 1020 genes upregulated and 402 genes downregulated (**Figure 3D**). Compared to the PBS group, interleukin-21 receptor-like (IL21R), interleukin-1 beta-like (IL1β), C-C motif chemokine 25-like (CCL25), C-C motif chemokine 20-like (CCL20) and other interleukin and chemokine-related genes were significantly upregulated. Cytoplasmic dynein 1 intermediate chain 1 (DYNC1I1), thrombospondin-4-B-like (THBS4B), thrombospondin-2 isoform X1 (THBS2), tubulin beta chain isoform X2 (TUBB), tubulin beta-4B chain-like (TUBB4B), cathepsin L1-like (CTSS), CD209 antigen-like protein E (CD209E) and other phagosome-related genes were significantly upregulated. Contactin-1 (CNTN1A), claudin-23-like (CLDN23), claudin-3-like (CLDN3**),** neuroligin-3 (NLGN3), neuroligin-4, X-linked (NLGN4X), contactin-associated protein 1 (CNTNAP1) and other cell adhesion molecules-related genes were significantly upregulated. Phosphatidylinositol 4,5-bisphosphate 3-kinase catalytic subunit alpha isoform-like (PIK3CA), phosphatidylinositol 3-kinase regulatory subunit beta-like (PIK3R2) and other phosphatidylinositol 3’-kinase-related genes were significantly upregulated. Double-stranded RNA-specific adenosine deaminase (ADAR), cyclic GMP-AMP synthase (MB21D1) and other cytosolic DNA-sensing-related genes were Significantly downregulated (**Table 3**).

**Table 3:** Summary of immune-related genes in the inactivated vaccine group compared to the PBS group at 7^th^ day post immunization.

Gene name	ID	Description	Log2 (FoldChange)	PValue
IL21R	ncbi_109627108	interleukin-21 receptor-like	1.213259565	4.82E-05
IL1β	ncbi_109641260	interleukin-1 beta-like	2.485190275	0.001848352
CCL25	ncbi_109625805	C-C motif chemokine 25-like	3.77792616	1.42E-05
CCL20	ncbi_109642248	C-C motif chemokine 20-like	2.321928095	0.025485066
DYNC1I1	ncbi_109631575	cytoplasmic dynein 1 intermediate chain 1	2.640289575	0.039754988
THBS4B	ncbi_109626438	thrombospondin-4-B-like	3.788246099	0.043135722
THBS2	ncbi_109636524	thrombospondin-2 isoform X1	3.475522895	0.014753712
TUBB	ncbi_109633300	tubulin beta chain isoform X2	3.065264296	0.00045301
TUBB4B	ncbi_109634278	tubulin beta-4B chain-like	1.616223249	0.031015525
CTSS	ncbi_109638800	cathepsin L1-like	1.230803026	0.000128832
CD209E	ncbi_109634808	CD209 antigen-like protein E	0.716422913	0.025319
CNTN1A	ncbi_109632653	contactin-1	2.835505962	0.003605804
CLDN23	ncbi_109627775	claudin-23-like	3.74723393	0.013697027
CLDN3	ncbi_109630440	claudin-3-like	3.829564906	0.000113599
NLGN3	ncbi_109625629	neuroligin-3	3.176877762	0.006897455
NLGN4X	ncbi_109640043	neuroligin-4, X-linked	3.040414268	0.004256591
CNTNAP1	ncbi_109634287	contactin-associated protein 1	2.72631835	0.018515565
PIK3CA	ncbi_109642929	phosphatidylinositol 4,5-bisphosphate 3-kinase catalytic subunit alpha isoform-like	2.357552005	0.028189952
PIK3R2	ncbi_109623868	phosphatidylinositol 3-kinase regulatory subunit beta-like	1.518337769	0.019644222
ADAR	ncbi_109633429	double-stranded RNA-specific adenosine deaminase	-0.68558248	0.001415993
MB21D1	ncbi_109631848	cyclic GMP-AMP synthase	-0.67861608	0.016658127

**Figure 4:**
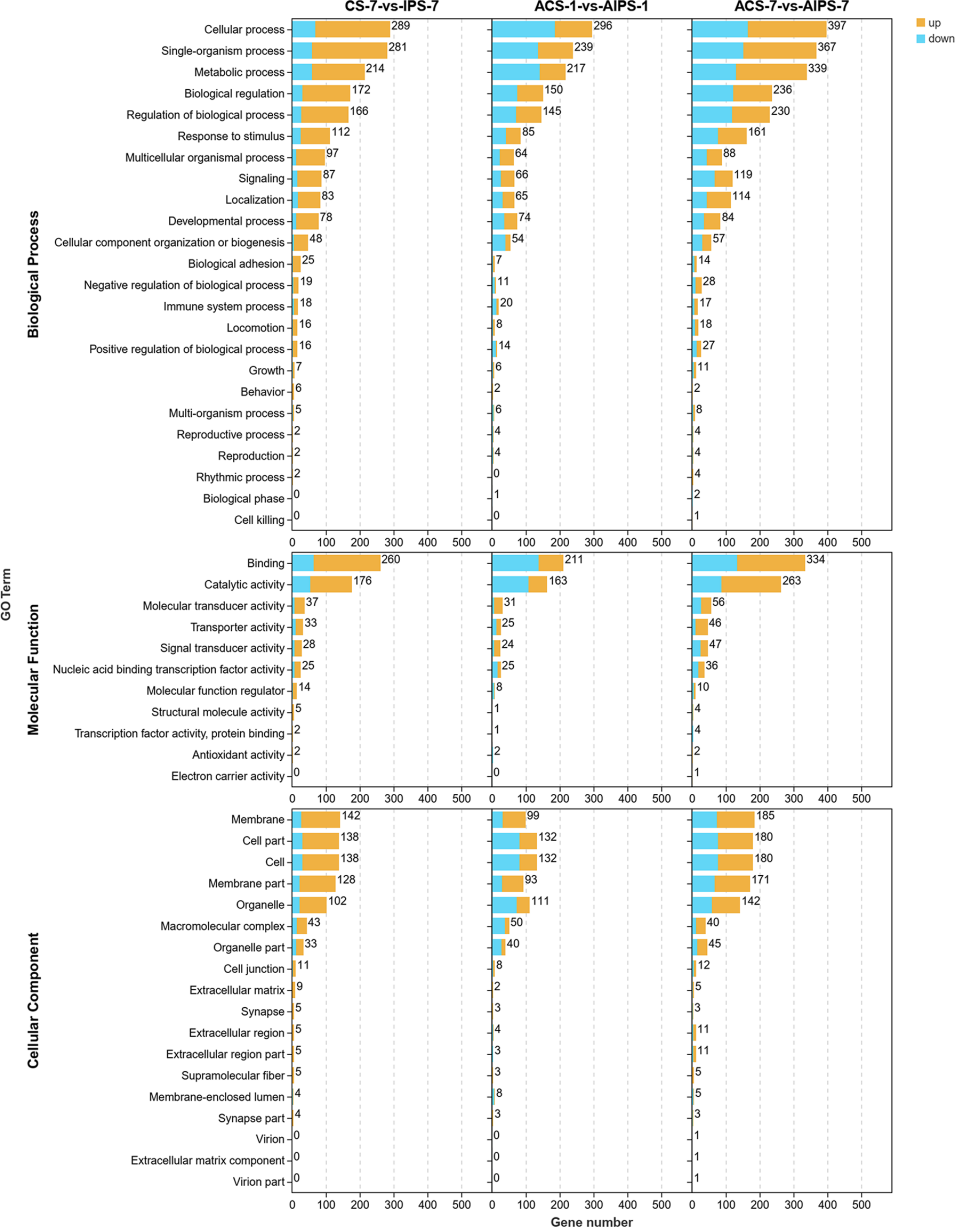
Gene ontology (GO) enrichment analysis of DEGs in the inactivated vaccine group compared to the PBS group at 7^th^ post immunization day and 1^st^ and 7^th^ post challenge day. 3-level GO annotations are distributed in three categories (biological process, molecular function and cellular component). Yellow represents up-regulated expression and blue represents down-regulated expression.

**Figure 5:**
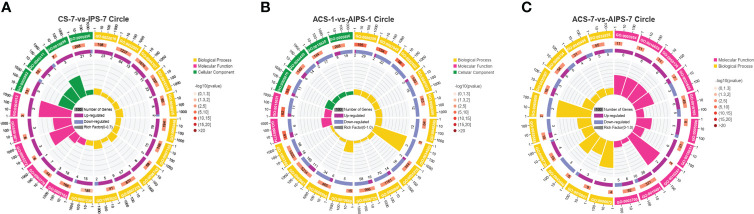
GO enrichment circle plot of Top 20 GO terms at 7^th^ post immunization day **(A)** and 1^st^ **(B)** and 7^th^ **(C)** post challenge day (*p* < 0.05). The first circle is the ID of the GO term, yellow represents the biological process, pink represents the molecular function, and green represents the cellular component. The second circle is the number of the GO term in the background genes and the *p* value. The third circle shows the proportion of up-regulated (deep purple) and down-regulated (blue) genes. The fourth circle shows the Rich Factor value of each GO term (Rich Factor value is the number of differential genes divided by the number of background genes in the GO term).

GO functional analysis showed that DEGs were involved in biological process (BP), molecular function (MF) and cellular component (CC) after immunization (**Figure 4**). Top 20 GO terms of DEGs enrichment were shown according to *p* < 0.05, with some GO terms associated with immunization. DEGs were mainly enriched in receptor binding and actin binding of MF; cytoskeleton, extrinsic component of membrane and intermediate filament cytoskeleton of CC; biological adhesion and multicellular organismal process of BP (**Figure 5A**, **Table 4**).

**Table 4:** ID, description, class and specific P-value of Top 20 GO terms at 7^th^ post immunization day.

ID	Description	Class	P value
GO:0005102	receptor binding	Molecular Function	0.000527
GO:0005882	intermediate filament	Cellular Component	0.005197
GO:0045111	intermediate filament cytoskeleton	Cellular Component	0.005197
GO:0019898	extrinsic component of membrane	Cellular Component	0.005656
GO:0005856	cytoskeleton	Cellular Component	0.005944
GO:0022610	biological adhesion	Biological Process	0.000198
GO:0003779	actin binding	Molecular Function	0.00324
GO:0005272	sodium channel activity	Molecular Function	0.003345
GO:0032501	multicellular organismal process	Biological Process	0.000873
GO:0044707	single-multicellular organism process	Biological Process	0.001025
GO:0016310	phosphorylation	Biological Process	0.00132
GO:0001871	pattern binding	Molecular Function	0.008587
GO:0004312	fatty acid synthase activity	Molecular Function	0.009584
GO:0008015	blood circulation	Biological Process	0.004093
GO:0006468	protein phosphorylation	Biological Process	0.004379
GO:0007155	cell adhesion	Biological Process	0.004788
GO:0098742	cell-cell adhesion *via* plasma-membrane adhesion molecules	Biological Process	0.00598
GO:0007275	multicellular organism development	Biological Process	0.008496
GO:1903522	regulation of blood circulation	Biological Process	0.008771
GO:0051239	regulation of multicellular organismal process	Biological Process	0.011304

**Figure 6:**
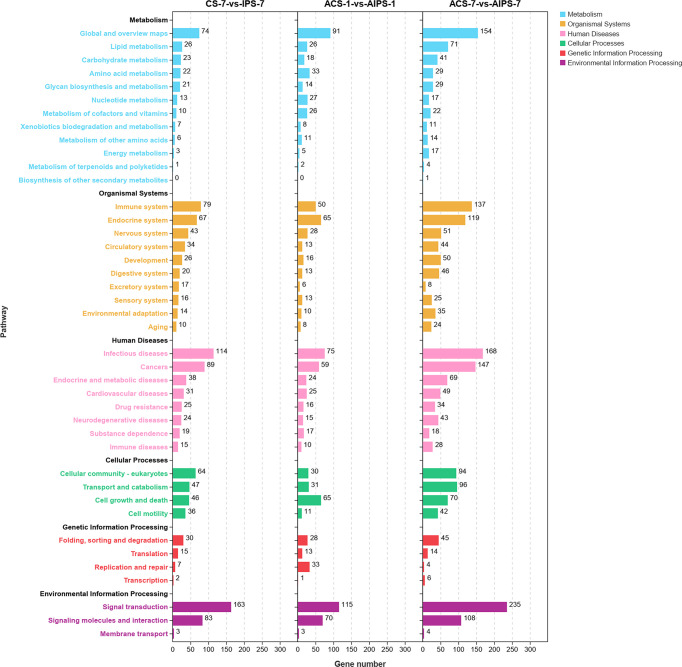
Kyoto Encyclopedia of Genes and Genomes (KEGG) enrichment analysis of DEGs at 7^th^ post immunization day and 1^st^ and 7^th^ post challenge day. The enriched pathways were counted in terms of level 1 and 2 classifications.

**Figure 7:**
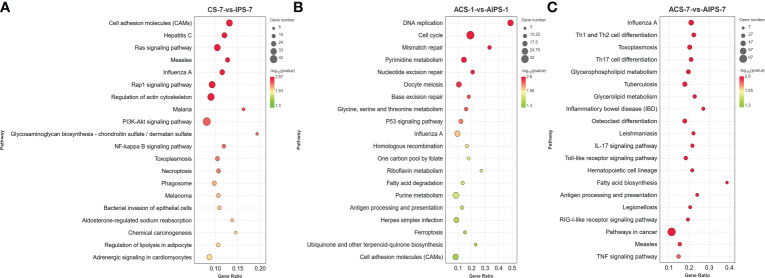
KEGG enrichment bubble plot of Top 20 pathways between PBS and inactivated vaccine groups at 7^th^ post immunization day **(A)** and 1^st^ **(B)** and 7^th^ **(C)** post challenge day (*p* < 0.05).

DEGs were enriched to six branches in KEGG (**Figure 6**), and bubble plots were used to demonstrate the top 20 signaling pathways (*p* < 0.05). At 7^th^ post immunization day, DEGs were significantly enriched to Phagosome, Cell adhesion molecules (CAMs), PI3K-Akt signaling pathway and NF-kappa B signaling pathway (**Figure 7A**).

### Early transcriptomic responses in the spleen of flounder challenged with *E. tarda*

On the first day after five weeks of immunization with *E. tarda* infection, 1210 DEGs (620 up-regulated genes and 590 down-regulated genes) were identified in the inactivated vaccine group compared with PBS group (**Figures 3B, D** **)**. Toll-like receptor 3 (TLR3), toll-like receptor 7 (TLR7), toll-like receptor 8 (TLR8), toll/interleukin-1 receptor domain-containing adapter protein isoform X1 (TIRAP) and other toll-like receptor-related genes were significantly upregulated. Junctional adhesion molecule B-like (JAM2), cadherin-4-like isoform X1 (CDH4), neural cell adhesion molecule 1-like isoform X1 (NCAM1), contactin-1a-like (CNTN1A) and other cell adhesion molecules-related genes were significantly upregulated. Inhibin beta B chain-like (INHBB), C-X-C chemokine receptor type 4-like (CXCR4), interleukin-21 receptor-like (IL21R), bone morphogenetic protein receptor type-1B isoform X1 (BMPR1B) and other Cytokine-cytokine receptor-related genes were significantly upregulated. Rano class II histocompatibility antigen, A beta chain-like (RT1-B), H-2 class II histocompatibility antigen, A-U alpha chain-like (RT1-Ba) and other Antigen processing and presentation-related genes were significantly upregulated. DNA replication ATP-dependent helicase/nuclease DNA2 isoform X1 (DNA2), replication factor C subunit 5 (RFC5), replication factor C subunit 2 (RFC2) and other cell cycle-related genes were significantly downregulated. G2 and S phase-expressed protein 1 isoform X1 (GTSE1), serine/threonine-protein kinase Chk1 (CHEK1), cyclin-G2-like (CCNG2) and other p53-related genes were significantly downregulated (**Table 5**).

**Table 5:** Summary of immune-related genes in the inactivated vaccine group compared to the PBS group at 1^st^ post challenge day.

Gene name	ID	Description	Log2 (FoldChange)	PValue
TLR3	ncbi_109641908	toll-like receptor 3	1.165898316	3.13E-06
TLR7	ncbi_109631070	toll-like receptor 7	1.439806437	1.30E-08
TLR8	ncbi_109631071	toll-like receptor 8	0.640640522	0.023441584
TIRAP	ncbi_109634523	toll/interleukin-1 receptor domain-containing adapter protein isoform X1	0.794222571	0.015091135
JAM2	ncbi_109631164	junctional adhesion molecule B-like	6.936441641	3.41E-05
CDH4	ncbi_109628880	cadherin-4-like isoform X1	1.854149134	0.014162315
NCAM1	ncbi_109632006	neural cell adhesion molecule 1-like isoform X1	1.784131965	0.000125865
CNTN1A	ncbi_109624594	contactin-1a-like	1.438370003	0.027450591
INHBB	ncbi_109637318	inhibin beta B chain-like	7.691161905	0.00451912
CXCR4	ncbi_109647982	C-X-C chemokine receptor type 4-like	3.934112064	0.000209948
IL21R	ncbi_109627108	interleukin-21 receptor-like	1.570552058	0.004386107
BMPR1B	ncbi_109638193	bone morphogenetic protein receptor type-1B isoform X1	1.304854582	0.010382372
RT1-B	ncbi_109633037	rano class II histocompatibility antigen, A beta chain-like	1.699003795	0.001496312
RT1-BA	ncbi_109633417	H-2 class II histocompatibility antigen, A-U alpha chain-like	1.42949101	0.003743397
DNA2	ncbi_109636867	DNA replication ATP-dependent helicase/nuclease DNA2 isoform X1	-0.735787403	0.012755301
RFC5	ncbi_109637136	replication factor C subunit 5	-0.629558444	0.005990157
RFC2	ncbi_109641693	replication factor C subunit 2	-0.910283724	1.77E-06
GTSE1	ncbi_109624619	G2 and S phase-expressed protein 1 isoform X1	-1.002573944	0.004375909
CHEK1	ncbi_109628292	serine/threonine-protein kinase Chk1	-0.625082809	0.026996525
CCNG2	ncbi_109646257	cyclin-G2-like	-0.680328178	0.000142464

GO functional analysis showed that at 1^st^ day post challenge, DEGs were mainly enriched to motor activity and tubulin binding of MF; cytoskeleton and microtubule cytoskeleton of CC; DNA metabolic process, cell cycle, and cell cycle process of BP (*p* < 0.05) (**Figure 5B**, **Table 6**).

**Table 6:** ID, description, class and specific P-value of Top 20 GO terms at 1^st^ post challenge day.

ID	Description	Class	P value
GO:0006259	DNA metabolic process	Biological Process	0
GO:0003774	motor activity	Molecular Function	0
GO:0005875	microtubule associated complex	Cellular Component	0.000002
GO:0007049	cell cycle	Biological Process	0.000001
GO:0015631	tubulin binding	Molecular Function	0.000007
GO:0044430	cytoskeletal part	Cellular Component	0.000032
GO:0022402	cell cycle process	Biological Process	0.000006
GO:0015630	microtubule cytoskeleton	Cellular Component	0.000065
GO:0007017	microtubule-based process	Biological Process	0.000012
GO:0005856	cytoskeleton	Cellular Component	0.00024
GO:0000280	nuclear division	Biological Process	0.000075
GO:0048285	organelle fission	Biological Process	0.000089
GO:0034502	protein localization to chromosome	Biological Process	0.000138
GO:0051276	chromosome organization	Biological Process	0.000427
GO:0006807	nitrogen compound metabolic process	Biological Process	0.000582
GO:0006725	cellular aromatic compound metabolic process	Biological Process	0.000622
GO:0015669	gas transport	Biological Process	0.000707
GO:0006996	organelle organization	Biological Process	0.00093
GO:0009987	cellular process	Biological Process	0.001137
GO:1901360	organic cyclic compound metabolic process	Biological Process	0.001235

KEGG functional analysis showed that DEGs were significantly enriched to Antigen processing and presentation, Cell adhesion molecules (CAMs), p53 signaling pathway (*p* < 0.05) (**Figure 7B**).

### Late transcriptomic responses in the spleen of flounder challenged with *E. tarda*

On the seventh day after five weeks of immunization with *E. tarda* infection, compared with the PBS group, 1106 genes were up-regulated and 823 genes were down-regulated (**Figures 3C, D**).

Toll-like receptor 7 (TLR7), signal transducer and activator of transcription 1-alpha/beta-like isoform X1 (STAT1), toll-like receptor 5 (TLR5) and other Toll-like receptor-related genes were significantly upregulated. Interleukin-12 receptor subunit beta-2-like (ILL2RB2), interleukin-12 subunit beta-like (IL12B), protein jagged-2-like (JAG2), interleukin-6 (IL6), interleukin-6 receptor subunit alpha-like (IL6R), heat shock protein HSP 90-alpha (HSP90A.1) and other T cell differentiation-related genes were significantly upregulated. Fibroblast growth factor 1 (FGF1), lysophosphatidic acid receptor 3 (LPAR3), lysophosphatidic acid receptor 4 (LPAR4), laminin subunit beta-1(LAMB1), integrin beta-4 isoform X1 (ITGB4) and other PI3K-Akt signaling pathway-related genes were significantly upregulated. Claudin-3-like (CLDN3), E-selectin (SELE), L-selectin-like (SELL) and other Cell adhesion molecules-related genes were significantly upregulated. Mitogen-activated protein kinase 14A-like isoform X2 (MAPK14A), mitogen-activated protein kinase 8-like isoform X1 (MAPK8) and other MAPK-related genes were significantly downregulated (**Table 7**).

**Table 7:** Summary of immune-related genes in the inactivated vaccine group compared to the PBS group at 7^th^ post challenge day.

Gene name	ID	Description	Log2 (FoldChange)	PValue
TLR7	ncbi_109631070	toll-like receptor 7	1.260077653	0.007232326
STAT1	ncbi_109640914	signal transducer and activator of transcription 1-alpha/beta-like isoform X1	1.103908424	1.16E-09
TLR5	ncbi_109643067	toll-like receptor 5	1.009899047	0.01759484
IL12RB2	ncbi_109625390	interleukin-12 receptor subunit beta-2-like	2.231325546	0.018502643
IL12B	ncbi_109636980	interleukin-12 subunit beta-like	2.127755547	0.03061254
JAG2	ncbi_109645635	protein jagged-2-like	0.753996675	0.004852413
IL6	ncbi_109631714	interleukin-6	9.120669887	0.01019172
IL6R	ncbi_109633010	interleukin-6 receptor subunit alpha-like	0.701641389	0.030149799
HSP90A.1	ncbi_109632540	heat shock protein HSP 90-alpha	1.50077805	6.78E-08
FGF1	ncbi_109635763	fibroblast growth factor 1	1.919829651	0.046466637
LPAR3	ncbi_109625857	lysophosphatidic acid receptor 3	1.460125389	9.64E-05
LPAR4	ncbi_109630081	lysophosphatidic acid receptor 4	1.259106188	9.13E-05
LAMB1	ncbi_109642034	laminin subunit beta-1	1.245061497	9.77E-07
ITGB4	ncbi_109627076	integrin beta-4 isoform X1	0.976370254	0.039128664
CLDN3	ncbi_109630427	claudin-3-like	2.611434712	0.042749421
SELE	ncbi_109625902	E-selectin	0.845011148	0.048004897
SELL	ncbi_109625903	L-selectin-like	0.823798588	0.010752208
MAPK14A	ncbi_109630822	mitogen-activated protein kinase 14A-like isoform X2	-0.602102745	0.011441485
MAPK8	ncbi_109633007	mitogen-activated protein kinase 8-like isoform X1	-0.633336555	0.018499353

GO functional analysis showed that DEGs were mainly enriched to NADH dehydrogenase activity, G-protein coupled nucleotide receptor activity and iron ion binding of MF, glycerol-3-phosphate metabolic process and endothelial cell differentiation of BP (**Figure 5C**, **Table 8**).

**Table 8:** ID, description, class and specific P-value of Top 20 GO terms at 7^th^ post challenge day.

ID	Description	Class	P value
GO:0003954	NADH dehydrogenase activity	Molecular Function	0.000082
GO:0016655	oxidoreductase activity, acting on NAD(P)H, quinone or similar compound as acceptor	Molecular Function	0.000082
GO:0050136	NADH dehydrogenase (quinone) activity	Molecular Function	0.000082
GO:0001608	G-protein coupled nucleotide receptor activity	Molecular Function	0.001157
GO:0016705	oxidoreductase activity, acting on paired donors, with incorporation or reduction of molecular oxygen	Molecular Function	0.001347
GO:0005506	iron ion binding	Molecular Function	0.003659
GO:0016651	oxidoreductase activity, acting on NAD(P)H	Molecular Function	0.004839
GO:0016297	acyl-[acyl-carrier-protein] hydrolase activity	Molecular Function	0.006364
GO:0016491	oxidoreductase activity	Molecular Function	0.008463
GO:0003700	transcription factor activity, sequence-specific DNA binding	Molecular Function	0.009143
GO:0006072	glycerol-3-phosphate metabolic process	Biological Process	0.00184
GO:0070997	neuron death	Biological Process	0.003505
GO:0045446	endothelial cell differentiation	Biological Process	0.004331
GO:0006739	NADP metabolic process	Biological Process	0.004632
GO:0035588	G-protein coupled purinergic receptor signaling pathway	Biological Process	0.005481
GO:0060850	regulation of transcription involved in cell fate commitment	Biological Process	0.006216
GO:0035587	purinergic receptor signaling pathway	Biological Process	0.006307
GO:0008154	actin polymerization or depolymerization	Biological Process	0.008692
GO:0046496	nicotinamide nucleotide metabolic process	Biological Process	0.009081
GO:0032535	regulation of cellular component size	Biological Process	0.009897

KEGG functional analysis showed that DEGs were significantly enriched to Th1 and Th2 cell differentiation, Th17 cell differentiation, IL-17 signaling pathway, Antigen processing and presentation, Toll-like receptor signaling pathway and RIG-I-like receptor signaling pathway, which are closely related to immunity (**Figure 7C**).

### Compared with immunization, the protection of vaccine on flounder after challenge

**Figure 8:**
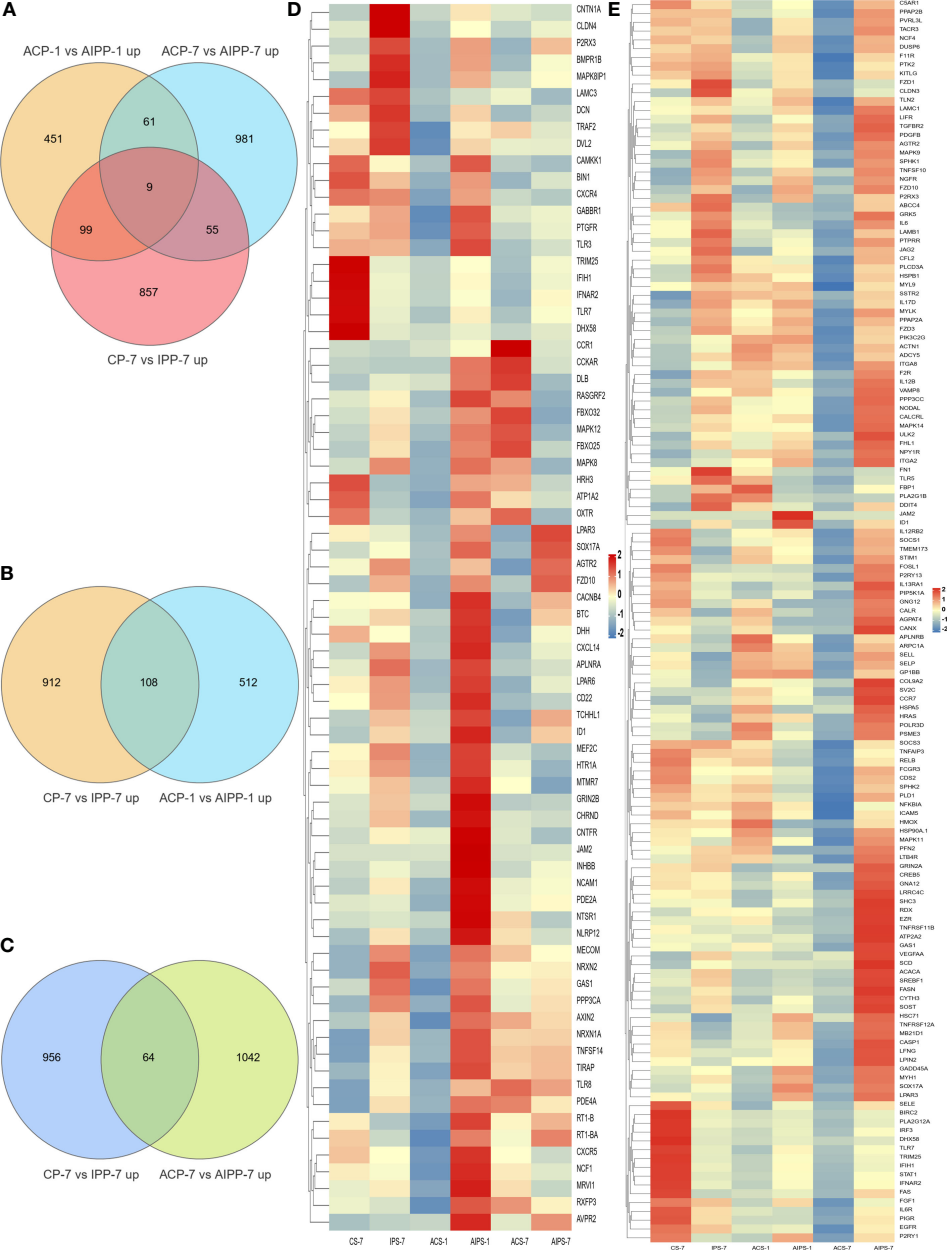
Venn diagram of upregulated genes between the PBS and inactivated vaccine groups at the three time points **(A)**. Venn diagram of genes upregulated between the seventh day after immunization and the first day after challenge **(B)** and heat map of 73 immune-related genes specifically upregulated on the first day after challenge between the PBS and inactivated vaccine groups at the three time points **(D)**. Venn diagram of genes upregulated between day 7 after immunization and day 7 after challenge **(C)** and heat map of 141 immune-associated genes specifically upregulated on day 7 after challenge in the PBS and inactivated vaccine groups at three time points **(E)**. The color shades in the heat map represent the gene expression levels. Closer to red indicates greater expression and closer to blue less expression.

**Figure 9:**
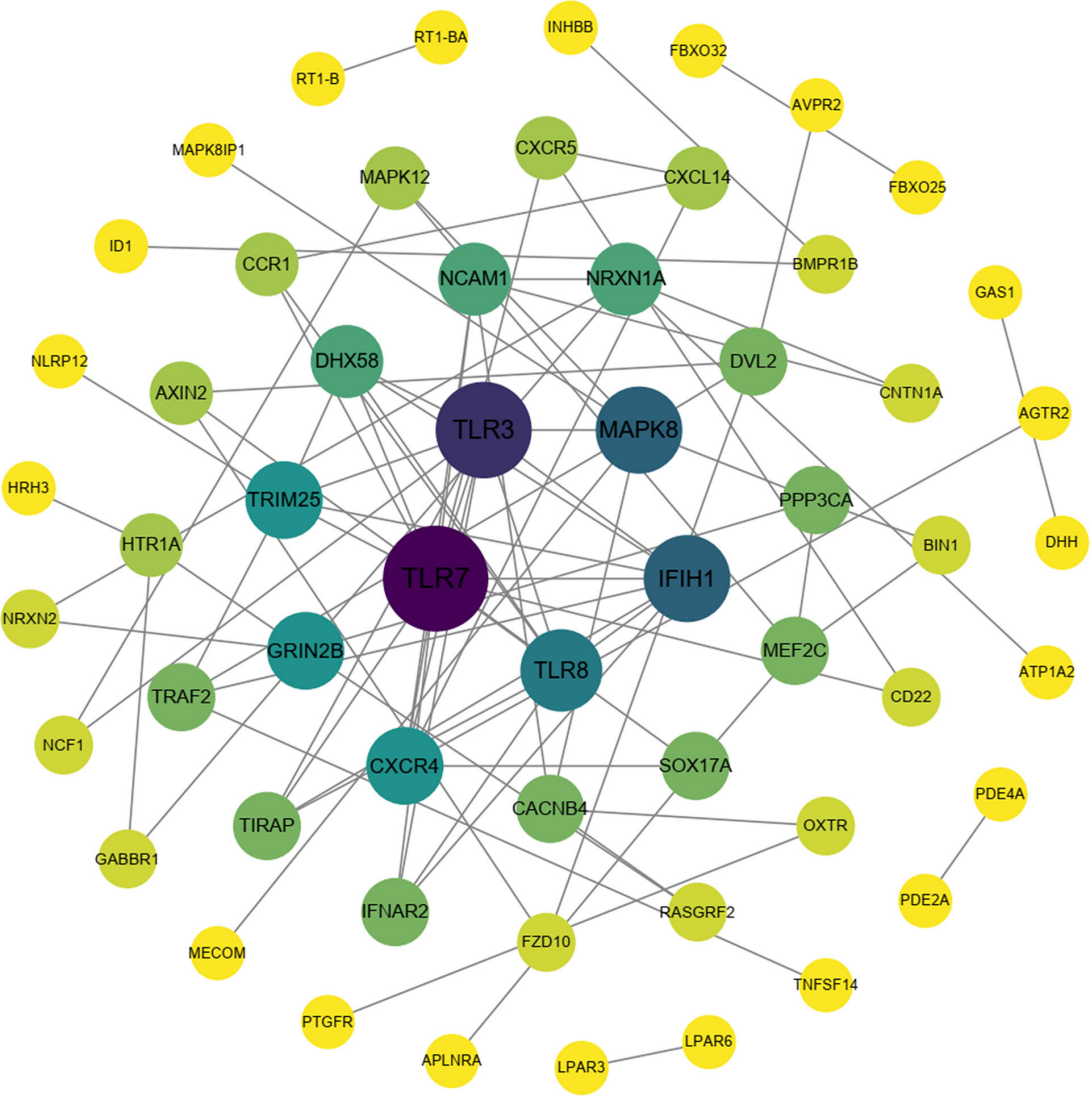
Protein interaction network analysis of immune-related upregulated genes on the first day after challenge.

**Figure 10:**
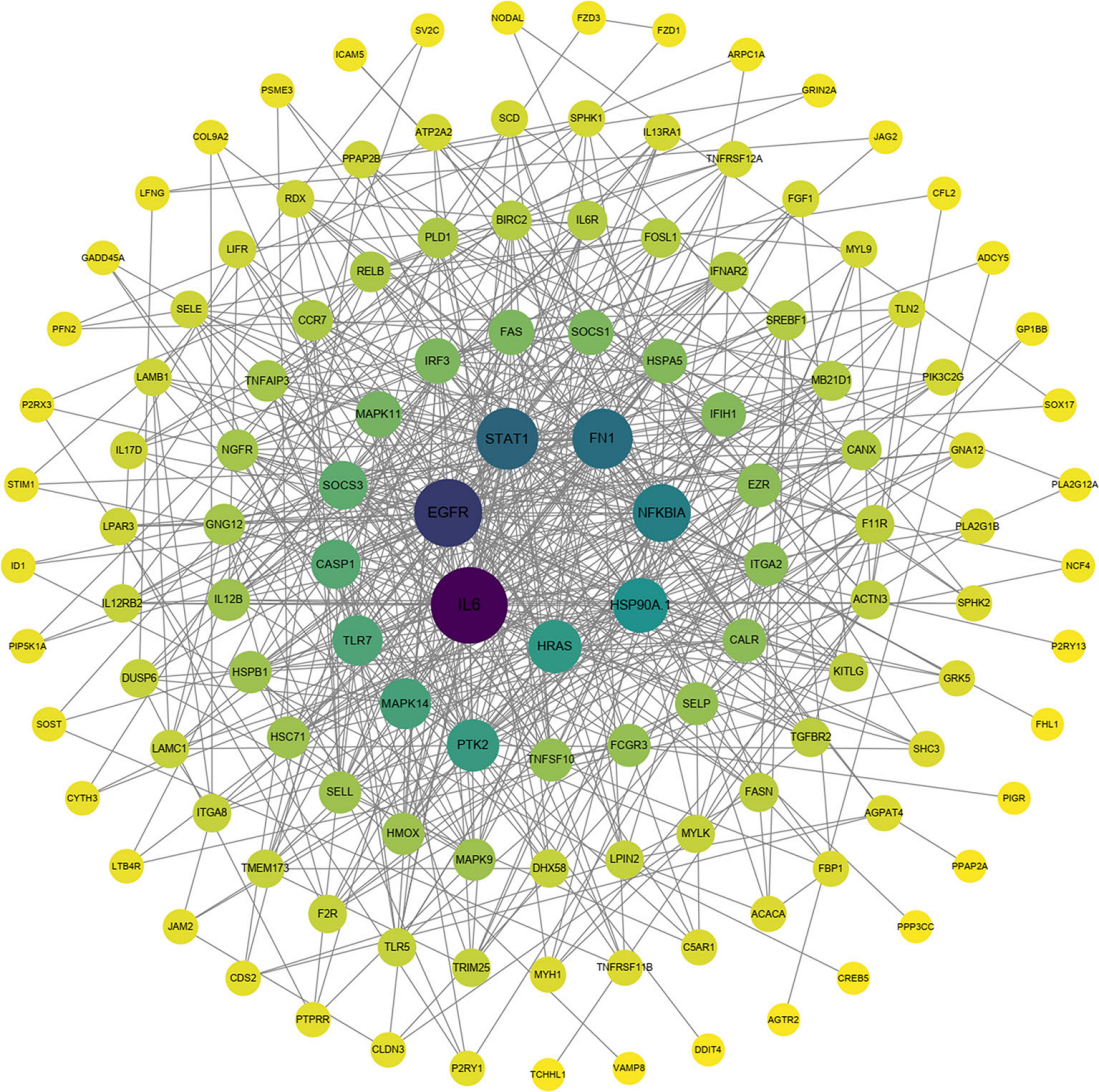
Protein interaction network analysis of immune-related upregulated genes on day 7 after challenge.

To explore the protection of vaccine after challenge compared to immunization. Venn diagram was used to show the common and specific profiles of upregulated DEGs at three time points. A specific 512 DEGs were upregulated at 1^st^ day post challenge compared to 7^th^ day post immunization (**Figure 8B**). Immune-related 73 DEGs were used to construct protein-protein interaction networks (**Table S1**). Their expression levels at three time points are represented by heat map (**Figure 8D**). 57 DEGs showed interactive network relationships. 11 hub genes (TLR7, TLR3, CXCR4, TLR8 etc) were identified in the network according to the number of node connections, most of which belong to the Toll-like receptor signaling pathway (**Figure 9**, **Table 9**). A specific 1042 DEGs were upregulated at 7^th^ day post challenge compared to 7^th^ day post immunization (**Figure 8C**). Immune-related 141 DEGs were used to construct protein-protein interaction networks (**Table S2**). Their expression levels at three time points are also represented by heat map (**Figure 8E**). 127 DEGs showed interactive network relationships. 30 hub genes (IL6, STAT1, HSP90A.1, TLR7, IL12B etc) were identified in the network according to the number of node connections (**Figure 10**, **Table 10**). In addition, a total of 9 DEGs were upregulated at the three time points. The immune-related DEGs were IL21R, Lpar4 (**Figure 8A**).

**Table 9:** Description and degree of connectivity of the 11 immune-associated upregulated hub genes on the first day after challenge.

Hub Gene	ID	Description	Degree
TLR7	ncbi_109631070	toll-like receptor 7	12
TLR3	ncbi_109641908	toll-like receptor 3	10
IFIH1	ncbi_109631067	interferon-induced helicase C domain-containing protein 1	8
MAPK8	ncbi_109633007	mitogen-activated protein kinase 8-like isoform X1	8
TLR8	ncbi_109631071	toll-like receptor 8	7
CXCR4	ncbi_109647982	C-X-C chemokine receptor type 4-like	6
GRIN2B	ncbi_109627266	glutamate receptor ionotropic, NMDA 2B-like isoform X1	6
TRIM25	ncbi_109627517	E3 ubiquitin/ISG15 ligase TRIM25-like	6
DHX58	ncbi_109634039	probable ATP-dependent RNA helicase DHX58	5
NCAM1	ncbi_109632006	neural cell adhesion molecule 1-like isoform X1	5
NRXN1A	ncbi_109627041	neurexin-1a-like isoform X1	5

**Table 10:** Description and degree of connectivity of the 30 immune-associated upregulated hub genes on day 7 after challenge.

Hub Gene	ID	Description	Degree
IL6	ncbi_109631714	interleukin-6	57
EGFR	ncbi_109646379	epidermal growth factor receptor	46
STAT1	ncbi_109640914	signal transducer and activator of transcription 1-alpha/beta-like isoform X1	38
FN1	ncbi_109624813	fibronectin-like	36
NFKBIA	ncbi_109632273	NF-kappa-B inhibitor alpha-like	33
HSP90A.1	ncbi_109632540	heat shock protein HSP 90-alpha	29
HRAS	ncbi_109641467	GTPase HRas-like	27
PTK2	ncbi_109638831	focal adhesion kinase 1 isoform X2	26
MAPK14	ncbi_109632714	mitogen-activated protein kinase 14-like	24
TLR7	ncbi_109631070	toll-like receptor 7	23
CASP1	ncbi_109630250	caspase-1-like isoform X1	22
SOCS3	ncbi_109633948	suppressor of cytokine signaling 3	21
MAPK11	ncbi_109632714	mitogen-activated protein kinase 14-like	18
FAS	ncbi_109631170	tumor necrosis factor receptor superfamily member 6-like isoform X1	17
IRF3	ncbi_109642328	interferon regulatory factor 3-like	17
SOCS1	ncbi_109645254	suppressor of cytokine signaling 1	17
HSPA5	ncbi_109626157	78 kDa glucose-regulated protein	16
IFIH1	ncbi_109631067	interferon-induced helicase C domain-containing protein 1	16
CALR	ncbi_109645752	calreticulin-like	15
EZR	ncbi_109647436	ezrin-like	15
ITGA2	ncbi_109636955	integrin alpha-2	15
FCGR3	ncbi_109630247	low affinity immunoglobulin gamma Fc region receptor III-like	14
TNFSF10	ncbi_109645712	tumor necrosis factor ligand superfamily member 10-like	14
SELP	ncbi_109648028	P-selectin-like	14
HMOX	ncbi_109627701	heme oxygenase-like	13
HSC71	ncbi_109628432	heat shock cognate 71 kDa protein	13
HSPB1	ncbi_109628310	heat shock protein beta-1	13
IL12B	ncbi_109636980	interleukin-12 subunit beta-like	13
MAPK9	ncbi_109626552	mitogen-activated protein kinase 9-like	13
SELL	ncbi_109625903	L-selectin-like	13

### qRT-PCR validation of transcriptomic immune-related genes

**Figure 11:**
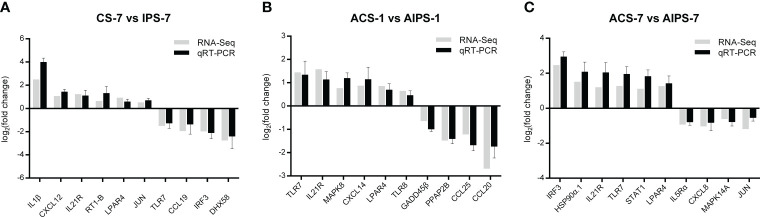
Comparison of gene expression levels between quantitative real-time polymerase chain reaction (qRT-PCR) and transcriptome sequencing at 7^th^ post immunization day **(A)** and 1^st^ **(B)** and 7^th^ **(C)** post challenge day.

The expression of ten immune-related genes at each time point was randomly examined using qRT-PCR. At 7^th^ day post immunization, the expression of IL1β, CXCL12, IL21R, RT1-B and LPAR4 were upregulated, while the expression of TLR7, CCL19, IRF5 and DHX58 were down-regulated (**Figure 11A**). At 1^st^ day post challenge, the expressions of TLR7, IL21R, MAPK8, CXCL14, LPAR4 and TLR8 were up-regulated, while the expressions of GADD45β, PPAP2B, CCL25 and CCL20 expressions were down-regulated (**Figure 11B**). At 7^th^ day post challenge, the expressions of IRF5, HSP90α.1, IL21R, TLR7, STAT1 and LPAR4 were up-regulated, while the expressions of IL5Rα, CXCL8, MAPK14A and JUN were down-regulated (**Figure 11C**). The expression levels of immune-related genes were basically consistent with the transcriptome results at the three time points, indicating that the sequencing results were accurate and reliable.

## Discussion

Immune response induced by vaccines is essential factor in protecting fish from pathogens (12, 48). In previous studies, flounder immunization with inactivated vaccines was effective against the challenge of *E. tarda* in terms of RPS, innate and specific immunity (13). The transcriptome at the genetic level can provide rich information on the mechanisms of the immune response in fish after vaccination and the status of the pathogen after infection. The spleen is the secondary immune organ with an important role in hematopoiesis and immunity. Here, flounder spleens were collected for RNA-Seq at 7^th^ day post immunization, 1^st^ and 7^th^ day post *E. tarda* challenge, respectively. DEGs were functionally annotated to explore immune response.

Vaccines trigger the body to produce the immune response in order to provide protection in case of pathogenic challenge. When vaccination is administered, the immune system makes a complex series of responses (3, 5, 49). First, many cytokines and chemokines are induced, causing an inflammatory response (50, 51). On the seventh day of marbled sleepy goby vaccination with inactivated iridovirus and rhabdovirus bivalent vaccine, pro-inflammatory cytokines such as IL-1β, IFN-γ, and IL-2R are upregulated to activate the innate immune response and, more importantly, to trigger a specific immune response (52). In study of IL-1β, IL-8, TNF-α and G-CSF as adjuvants for OmpV of *E. tarda* subunit vaccine, IL-1β and IL-8 were reported to significantly enhance serum antibodies and sIg^+^B lymphocytes, and the expression of genes (CD4-1, CD4-2, MHCIIα and IgM) related to cellular and humoral immunity (53, 54). Similarly, in this study, inflammatory and chemotaxis-related genes such as IL21R, IL1b, CCL25, and CCL20 were significantly upregulated at day 7 after immunization, activating cell recruitment and laying the foundation for triggering adaptive immunity. Phagocytosis is the process of host defense against pathogens. In innate immunity, macrophages, dendritic cells and neutrophils take up antigens through endocytosis. The antigen is then digested by lysosomes and the antigen signal is presented to the specific immune system (55). In adaptive immunity, B lymphocytes have been demonstrated to have phagocytic effect in fish. A previous study showed that B lymphocytes of dental flounder could phagocytose inactivated *Lactococcus lactis* (*L. lactis*). Transcriptome sequencing analysis of B lymphocytes after *L. lactis* stimulation showed that many DEGs were enriched to the phagocytic pathway. Further studies revealed the key role of Fc receptor (FcR) in regulating phagocytosis and bactericidal activity of B lymphocytes (56, 57). After 2 and 4 weeks of turbot (*Scophthalmus maximus*) inoculation with the bivalent inactivated bacteria vaccine *Aeromonas salmonicida* and *Vibrio scophthalmi*, Phage-associated genes such as Calreticulin (CALR), Antigen peptide transporter 1 (TAP1), and Integrin beta-3 (αVβ3) C-type mannose receptor 2 (MRC2) were upregulated in the kidney (33). Integrin beta-5-like (ITGB5), cyptoplasmic dynein 1 intermediate chain 1 (DYNCLI1), and thrombospondin-4-B-like (THBS4B) of the Phagosome pathway were also significantly enriched in this study, indicating that the early immune response underwent antigen processing. Cell adhesion molecules are involved in recognition between cells by means of ligand and receptor binding. In addition, in the immune response, it transmits signals for the antigen delivery process (58). In muscles around the injection site of flounder (*Paralichthys olivaceus*) vaccinated with VAA DNA vaccine, cell adhesion molecules enhance the local immune response by mediating the recruitment of immune cells to the site of inflammation (59). In addition, the PI3K-Akt signaling pathway is important node for signaling. It promotes cell proliferation, differentiation and anti-apoptosis after receiving extracellular signals (60). PIK3CD and PIK3R2 were identified as hub genes involved in the immune response in flounder gill infected with *E. tarda*. It was found that the expression of PIK3CD decreased continuously, while the expression of PIK3R2 increased and then decreased during infection. Combined with its transduction of antigenic signals, it affects the specific immune response, which is inhibited by *E. tarda* (61). In the present study, genes related to CAM (CNTN1A, CLDN23, CLDN3, NLGN3, NLGN4X) and PI3K family (PIK3R2, PIK3CA) were significantly upregulated after immunization.

At 1^st^ day post challenge, pattern recognition receptors (PRRs), such as Toll-like receptors, elicit the body’s immune response by binding to pathogen-associated molecular patterns (PAMPs) (62). Eleven TLR family members were identified in flounder (63). TLR3, TLR7, TLR8 and TIRAP were significantly upregulated in this study. In mammals, TLR3, TLR7 and TLR8, located in intracellular vesicles, are critical receptors for the recognition of viral nucleic acids in the antiviral response (64). TLR7 was upregulated in both head kidney and spleen within 48 h after tongue sole infection with *Pseudomonas fluorescence*, and knockdown of TLR7 resulted in significantly higher bacterial load in tissues than in control (65). In the intestine of black rockfish *Sebastes schlegelii* infected with *E. tarda*, TLR3 showed upregulation at 2h, 6h, 12h and 24h (66). The TLR family of bony fish has a more complex immune response to pathogens such as viruses and bacteria than that of mammals. In the protective response of vaccines, antigen delivery is the initiation of adaptive immunity. Antigen signals are presented to T cells, which can specifically bind and kill target cells or release cytokines to stimulate B cell proliferation and differentiation (67). Among them, MHC molecules expressed on antigen-presenting cells such as dendritic cells and B cells are the markers of presentation (68). MHC class I presents antigenic fragments to CD8^+^ T cells for killing of target cells by releasing cytotoxic particles (perforin and granzyme) (69, 70). MHC class II presents antigenic fragments to CD4^+^ T cells to achieve expanded and increased immune effector functions through synthesis and release of cytokines (71). The expression of MHC Iα, MHC IIα, CD4-1 and CD8α was significantly upregulated in immunized tissues when flounder was immunized with inactivated *E. tarda* vaccine. In addition, these genes were also significantly elevated in the spleen and head kidney after five weeks of immunization with *E. tarda* infection. This indicates that immunization activates both cellular and humoral immune responses (13, 47). Vaccinated Arctic Charr showed significant expression of TLR7 after infection with *Aeromonas salmonicida*, activating B cells and DC to produce IFN-α and triggering Th1 and CD8^+^ T cell responses to demonstrate the effect of vaccination (72). Antigen processing and presentation-related genes (TAP1, TAP2, ABCB9 and PSME2) were identified in flounder spleen erythrocytes infected with *E. tarda* for 24h (32). Full-length transcriptome sequencing was performed on several tissues (liver, kidney, intestine, skin, gill) involved in immune and metabolic processes in black rockfish (*Sebastes schlegelii*). Four immune-related genes annotated as H-2 class II histocompatibility antigens were mined (73). In the present study, RT1-B and RT1-Ba were identified. In addition, Cytokine-cytokine receptor interaction as well as adhesion molecules (CAMs) remained functional after *E. tarda* infection. Toll-like receptors form the initial barrier against pathogens by specifically recognizing pathogens. In addition, TLR signaling induces DCs to produce IL-1β, IL-6, IL-12, and chemotactic cytokines that regulate antigen-specific Th1 and Th2 cell differentiation, linking innate and adaptive immunity (74–77). Marbled sleepy goby vaccinated with inactivated iridovirus and rhabdovirus bivalent vaccine showed consistent upregulation of MHC I, CD8, TCR, MHC II, CD4 and IgM expression after 2 days of *Oxyeleotris marmoratus rhabdovirus* challenge, indicating rapid induction of cellular and humoral immunity (52). At 7^th^ day post challenge, TLR5, TLR7, il6, il1b, and IL12B were still highly expressed, suggesting that TLR may play a role in both early and late stages of infection. In mammals, stimulated by different antigens, CD4^+^ T lymphocytes differentiate into different cell subtypes (Th1, Th2, Th17 and Treg cells). This mechanism has also been demonstrated in teleost fish (78). Flounder were immunized with the NADP-dependent isocitrate dehydrogenase (IDH) subunit vaccine of *E. tarda*. The expression of Th1 and Th2 immune-related genes (IL-1β, TNF-α, IL-8, IL-6, NKEF, IFN-γ) was significantly increased. After infection with *E. tarda*, the bacterial load in the tissues was significantly reduced and the RPS reached 73.3%, which provided good protection against edwardsiellosis (46). In the present study, Th1 and Th2 cell differentiation, Th17 cell differentiation were significantly enriched, indicating that activation of cellular immunity plays an important role in vaccine protection against bacterial infection.

According to previous experiments, the immune response was initially activated at 7^th^ day post immunization (36, 79). The response of the organism to the pathogen is strong and rapid after infection, with 24 h being important time point. 73 immune-related genes were activated on day 1 after *E. tarda* challenge compared to day 7 after immunization. 11 hub gene were identified, in which TLR family members were more enriched. TLR7 acts in organelles such as endoplasmic reticulum and lysosomes, which recognize viral single-stranded RNA and induce IFN-α, cytokine and chemokine production (80). In addition, the Toll-IL-1 receptor domain interacts with the junctional protein MyD88 in the antimicrobial immune response, activating the downstream NF-κB signaling pathway and producing pro-inflammatory cytokines. The bacterial load in the tissues of tongue sole (*Cynoglossus semilaevis*) infected with *Pseudomonas fluorescence* TSS was significantly enhanced after knockdown of CsTLR7. The results suggest that CsTLR7 has a positive role in the clearance of bacterial pathogens (65). In mammals, TLR3 specifically recognizes viral double-stranded RNA (dsRNA) and triggers toll interleukin-I receptor domain (TIR) through the myeloid differentiation factor 88 (MyD88) non-dependent pathway, activating downstream type I interferon gene expression and the NF-κB signaling pathway to induce an antiviral response in the organism (81). High expression of TLR3 was detected in the spleen and head kidney of channel catfish infected with virulent *Edwardsiella ictalurid* (82). TLR3 showed upregulation in the intestine of black rockfish *Sebastes schlegelii* for 24 h after *Edwardsiella tarda* infection (66). In the present study, TLR3 expression was upregulated on the first day after infection, suggesting that TLR3 in fish also plays a role in resistance to bacterial infection. TLR8, which is highly homologous to TLR7, functions in the lysosome to recognize bacterial or viral single-stranded RNA (83). TLR8 expression was detected in mucosal tissues (skin, gill and intestine) of turbot after infection with *Vibrio anguillarum and Streptococcus iniae* (84). Based on their central position in the interaction network, TLRs may play the important function in the anti-infection response of the fish spleen.

141 immune-related genes were activated on day 7 after *E. tarda* challenge compared to day 7 after immunization. Inflammatory cytokines (IL6, IL12B, IL6R, IL12RB2, IL17D), transcription factors (STAT1, IRF3) showed the strong interaction according to the functional classification. IL-12, which is involved in Th1 differentiation, is produced by DCs. IL12B (p40) and IL-12A (p35) together encode IL12, which acts by inducing the production of IFN-γ. IL-12Rβ1 and IL-12Rβ2 form the IL-12R complex, which is mainly expressed by activated T cells and natural killer cell. IL-12 and IL-12R binding activates the JAK2/STAT4 pathway to increase IFN-γ production as well as induce shift of T cells to Th1 phenotype (85). Th1 cells secrete IFN-γ, TNF-β, IL-2, etc. to mediate cellular immunity, which effectively defends against infection by intracellular pathogens (86, 87). A previous study showed that T-bet is a transcription factor involved in the immune response of Th1 cells. The expression was significantly increased after *E. tarda* infection, which laterally corroborates the important role of Th1 cells in responding to pathogenic infections. Moreover, IFN-γ and IL-2 were able to upregulate T-bet expression and contribute to the differentiation of Th0 cells to Th1 cell type (88). On day 14 of immunization with inactivated iridovirus and rhabdovirus bivalent vaccine and on day 7 of *Oxyeleotris marmoratus iridovirus* infection, the expression of inflammatory cytokines such as IL-12 and IL-1β was upregulated in the spleen of marbled sleepy goby, realizing the inflammatory of the innate immune response and leading to the development of adaptive immunity (52). In the present study, IL-6 was the hub gene of the protective immune network at 7^th^ post challenge day. Previous studies have shown that significant upregulation of IL-6 was also detected after immunization of flounder with rIDH vaccine (46). IL-6 is pro-inflammatory cytokine produced mainly by macrophages and Th2 cells. IL-6 forms a complex with IL-6R, which binds to the membrane protein gp130, which activates intracellular signal transduction to function. It can promote the proliferation activation of T cells and the expression of IL-2 receptor on the surface of T cells, which further assists the proliferation and differentiation of B cells and the production of antibodies to participate in the humoral immune response (89, 90). A previous study showed that cyclosporine A (CsA) inhibited T lymphocyte expression by blocking activation of the transcription factor NFAT, and then inhibited B lymphocyte expression and antibody production. This suggests that T lymphocytes have an important regulatory role on B lymphocytes in the immune response (91, 92). In response to cytokines (IL-6, IFNγ) and growth factors (epidermal growth factor), STAT1 forms dimers that are transported to the nucleus to regulate apoptosis and the cell cycle (93). After *P. olivaceus* was infected with *E. tarda* for 8h and 48h, STAT1 was identified as hub gene in blood, gill, and kidney expression profiling (35, 61, 94). In the present study, IL21R was significantly upregulated at all three time points, corroborating its protective role in immunity and infection. IL21R is cytokine receptor for IL21. It is expressed on activated NK cells and belongs to the type I cytokine receptor. The receptor binds to IL21, leading to the activation of several downstream molecules (JAK1, JAK3, STAT1 and STAT3), while inducing the proliferation and differentiation of T cells, B cells and natural killer (NK) cells (95, 96).

The spleen, as an important lymphoid organ, is the main site of the immune response. Previous studies have found strong immune response in the spleen after flounder immunization or infection (13, 47, 97, 98). In the study of flounder immunized with inactivated *E. tarda* vaccine, the uptake of antigen and antigen presentation-related immune genes (MHC Iα, MHC IIα, CD4-1 and CD8α etc) were significantly elevated in the spleen. The immunohistochemical results of the spleen showed that CD4^+^ and CD8^+^ T lymphocyte were distributed around the melanocyte macrophage center (MMC). This fully demonstrates the important role of the spleen in capturing antigens, aggregating macrophages and lymphocyte populations, and presenting antigens to lymphocytes to activate the adaptive immunity (47, 97). Also in the study of immunized flounder infected with *E. tarda*, the immune response in the spleen varied significantly at the tissue, T/B lymphocyte and genetic levels and was the main tissue for monitoring the protective effect (13, 98). Keeping in line with previous studies, in this work, the spleen was sampled for transcriptome sequencing analysis after immunization with inactivated vaccine and infection with *E. tarda*. In terms of immune and protective responses, the strong immune response (cytokines, T- and B-cell related factors, etc.) in the spleen was also confirmed. In addition, in the immune system of fish, there are temporal differences in the production of cytokines, T/B lymphocytes, and antibodies, which are closely related to immune protection (12, 13). The overall immune response requires a multitemporal analysis, which is important for a comprehensive and detailed elucidation of vaccine immunization mechanism (99, 100). For example, the humoral immune response and the production of antibodies require a longer response time to be effective. IgM was significantly upregulated in marbled sleepy goby immunized with inactivated iridovirus and rhabdovirus bivalent vaccine for two weeks, indicating that the humoral immune response was activated (52). In the vaccine evaluation, antibodies were produced in fish during the immunization phase of two weeks to four weeks (12, 101, 102). The study of multiple time points of immune response is also worth doing to fully reveal the mechanism of fish vaccines.

## Conclusions

This study investigated the mechanism of immune protection in flounder inoculated with inactivated *E. tarda* vaccine. Transcriptome sequencing analysis of the flounder spleen was performed on the seventh day after immunization and on the first and seventh day after five weeks of immunization with *E. tarda* infection. 1422, 1210 and 1929 DEGs were identified, respectively, which were significantly enriched in immune-related pathways such as Toll-like receptor signaling pathway and Th1 and Th2 cell differentiation. In addition, immune-related hub genes were identified after *E. tarda* infection compared to after immunization, in which TLR family members (TLR3, TLR5, TLR7, TLR8), pro-inflammatory cytokines and their receptors (IL6, IL6R, IL12B, IL12RB2, IL17D) were the main regulators that exerted immune protection. These genes are closely associated with the recognition and presentation of pathogens and the activation of cellular immunity. This study analyzed the transcriptional profiles of flounder spleen after immunization and infection, providing basis for further elucidation of the immune protection mechanisms in flounder immunized with inactivated *E. tarda* vaccine.

## Data availability statement

The datasets presented in this study can be found in online repositories. The names of the repository/repositories and accession number(s) can be found in the article/**Supplementary Material**.

## Ethics statement

The animal study was reviewed and approved by the Institutional Animal Care and Use Committee of the Ocean University of China (permit number: 20150101).

## Author contributions

XW and JX contributed to the conception and design of this experiment, performed most of the experiments and statistical analysis, drafted and revised the manuscript. XT, HC, and XS participated in the design of the study, helped analyzed experiments and data. JX and WZ design the study, provided reagents, instruments and experiment space. All authors contributed to the article and approved the submitted version.

## Funding

This study was supported by the National Natural Science Foundation of China (32173005; 31730101; 31672684; 31672685), Shandong Provincial Natural Science Foundation (ZR2020KC025), the National Key Research and Development Program of China (2018YFD0900503).

## Conflict of interest

The authors declare that the research was conducted in the absence of any commercial or financial relationships that could be construed as a potential conflict of interest.

## Publisher’s note

All claims expressed in this article are solely those of the authors and do not necessarily represent those of their affiliated organizations, or those of the publisher, the editors and the reviewers. Any product that may be evaluated in this article, or claim that may be made by its manufacturer, is not guaranteed or endorsed by the publisher.
